# Supplementary material for: Predicting visual function by interpreting a neuronal wiring diagram
Source: Nature. 2024 Oct 2;634(8032):113–23. doi: 10.1038/s41586-024-07953-5 (PMC11446822; doi:10.1038/s41586-024-07953-5)
Supplement: Supplementary file 6 — CRF and ERF predictions for individual TmY4 and TmY9 cells. Analogous to Supplementary Data 3, but for TmY target types. Shown are the top four monosynaptic pathways, the strongest pathway passing through each of the top ten intermediary types (ranking from Extended Data Fig. 7), and the trisynaptic pathway Tm1–TmY–Dm3–TmY (see the section entitled Prediction of spatial normalization). [file 41586_2024_7953_MOESM6_ESM.zip › DataS4/TmY9qΓèÑ/720575940617648614.pdf]

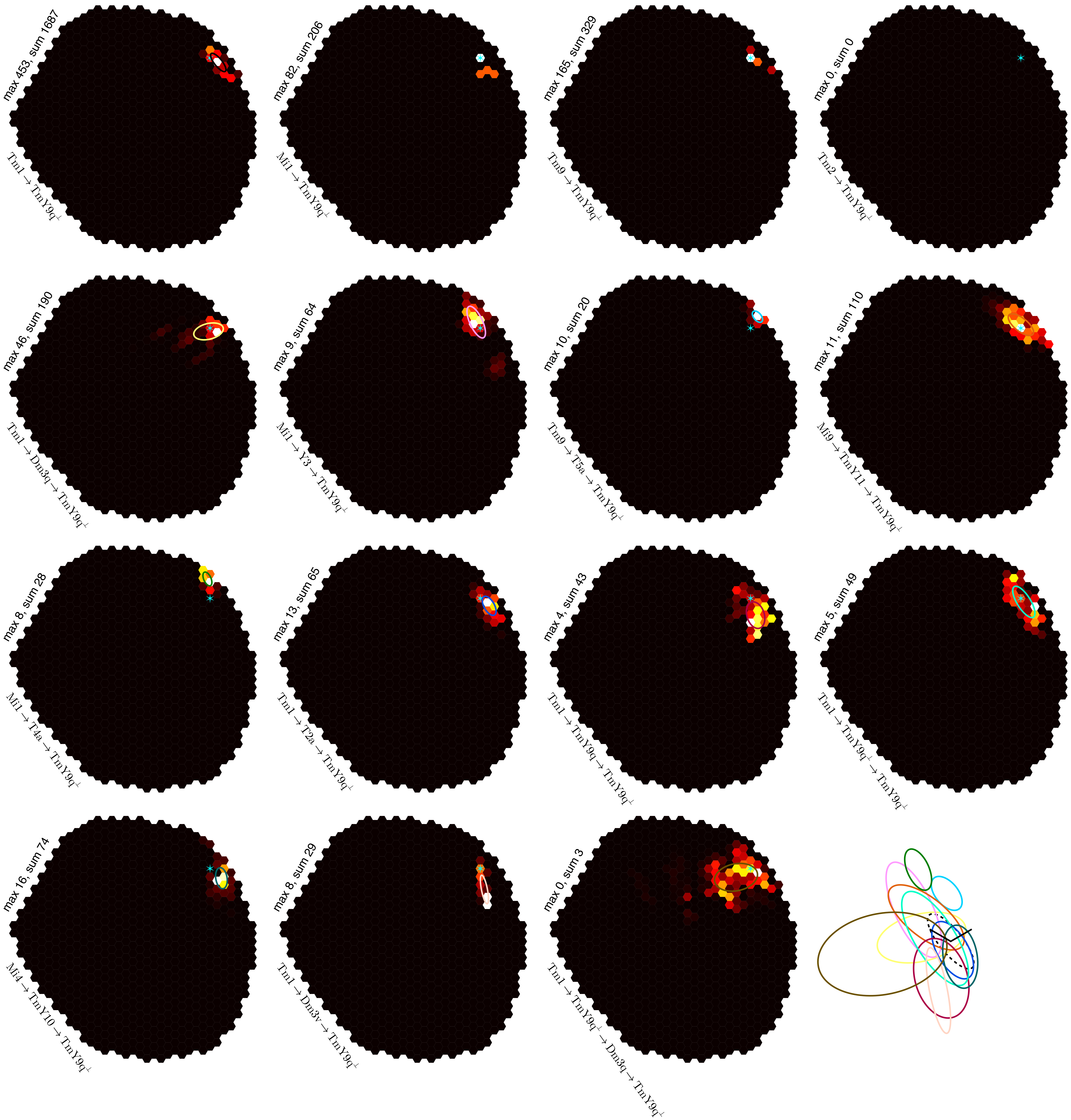
max 453, sum 1687
Tm1 → TmY9q⊥
max 82, sum 206
Mi1 → TmY9q⊥
max 165, sum 329
Tm9 → TmY9q⊥
max 0, sum 0
Tm2 → TmY9q⊥
max 46, sum 190
Tm1 → Dm3q → TmY9q⊥
max 9, sum 64
Mi1 → Y3 → TmY9q⊥
max 10, sum 20
Tm9 → T5a → TmY9q⊥
max 11, sum 110
Mi9 → TmY11 → TmY9q⊥
max 8, sum 28
Mi1 → T4a → TmY9q⊥
max 13, sum 65
Tm1 → T2a → TmY9q⊥
max 4, sum 43
Tm1 → TmY9q → TmY9q⊥
max 5, sum 49
Tm1 → TmY9q⊥ → TmY9q⊥
max 16, sum 74
Mi4 → TmY10 → TmY9q⊥
max 8, sum 29
Tm1 → Dm3v → TmY9q⊥
max 0, sum 3
Tm1 → TmY9q⊥ → Dm3q → TmY9q⊥
